# The Cyclopædia of Practical Medicine

**Published:** 1844-10

**Authors:** 


Art. IX.—*The Cyclopædia of Practical Medicine.* Edited by John Forbes, M.D., F.R.S., Alex. Tweedie, M.D., F.R.S., and John Conolly, M.D. Revised, with additions, by Robley Dunglison, M.D. Parts VIII., IX., X., and XI. Philadelphia: Lea & Blanchard. 1844.

These numbers of the Cyclopædia bring the work nearly to the end of the second volume, and embrace a great variety of subjects. The task of the publishers is nearly half completed. We hope the demand for the work is equal to its merits, of which there is no occasion that we should repeat the favorable opinion which we have more than once expressed. Y.

